# Dysregulated bidirectional epithelial-mesenchymal crosstalk: a core determinant of lung fibrosis progression

**DOI:** 10.1016/j.pccm.2024.02.001

**Published:** 2024-03

**Authors:** Liudi Yao, Zijian Xu, Donna E. Davies, Mark G. Jones, Yihua Wang

**Affiliations:** 1 Biological Sciences, Faculty of Environmental and Life Sciences, University of Southampton, Southampton SO17 1BJ, UK; 2 Clinical and Experimental Sciences, Faculty of Medicine, University of Southampton, Southampton SO16 6YD, UK; 3 Institute for Life Sciences, University of Southampton, Southampton SO17 1BJ, UK; 4 NIHR Southampton Biomedical Research Centre, University Hospital Southampton, Southampton SO16 6YD, UK

## Abstract

Progressive lung fibrosis is characterised by dysregulated extracellular matrix (ECM) homeostasis. Understanding of disease pathogenesis remains limited and has prevented the development of effective treatments. While an abnormal wound healing response is strongly implicated in lung fibrosis initiation, factors that determine why fibrosis progresses rather than regular tissue repair occurs are not fully explained. Within human lung fibrosis there is evidence of altered epithelial and mesenchymal lung populations as well as cells undergoing epithelial–mesenchymal transition (EMT), a dynamic and reversible biological process by which epithelial cells lose their cell polarity and down-regulate cadherin-mediated cell–cell adhesion to gain migratory properties. This review will focus upon the role of EMT and dysregulated epithelial-mesenchymal crosstalk in progressive lung fibrosis.

## 1 Idiopathic pulmonary fibrosis (IPF)

Fibrosis is a fundamental cause of morbidity and mortality worldwide, and prevalence is increasing because of the ageing population. Once considered rare it is now recognised that interstitial lung diseases (ILD) are present in 7% of the general population (age > 50), resulting in an increase in all-cause mortality (hazard ratio 2.7). This prevalence has further increased as a consequence of the COVID pandemic during which, we identified long-term persistent interstitial change following COVID pneumonitis ^1,2^. Idiopathic pulmonary fibrosis (IPF) is a specific type of chronic fibrotic ILD, characterised by a diagnosis of the usual interstitial pneumonia (UIP) pattern ^3–5^, affecting the air space between the alveolar epithelium and the capillary endothelium. Canonically, it is characterised by a disrupted balance of extracellular matrix (ECM) homeostasis, resulting in ECM deposition and lung parenchyma expansion ^6^. As a progressive pulmonary disorder, it is often asymptomatic during early disease stages. Over time, with considerable scarring within the lung, patients mainly suffered from dry cough and dyspnoea, resulting in poor survival ^3^. Once diagnosed with IPF, studies suggested that the median survival is between 2 and 3 years ^7,8^. Although some risk factors have been found, the pathogenesis of IPF remains unclear.

Canonically, IPF is proposed to occur as a consequence of abnormal wound healing responses. As IPF is proposed to be an epithelial-driven and myofibroblast-activated progressive deterioration process ^9^, improved understanding of the distinct role of these 2 populations is of high importance.

### 1.1 Epithelial cells in IPF

While micro-injuries and dysfunction of alveolar epithelium are strongly implicated in IPF initiation ^10^, factors determining why pathogenic ECM remodelling rather than regular wound resolution occurs remain poorly explained.

Altered phenotypes in alveolar epithelial cells (AECs) in IPF have been characterised ^11,12^. A major finding of a single-cell RNA sequencing (scRNA-seq) study published recently is the identification of a significant shift in epithelial cell phenotypes in the peripheral lung in pulmonary fibrosis (PF), including several previously unrecognized epithelial cell phenotypes. Notably, they found a pathologic epithelial cell population (KRT5^-^/KRT17^+^) that produces ECM and is highly enriched in PF lungs. Trajectory analysis suggested that this phenotype evolve from a transitional stage of Type II alveolar epithelial (ATII) cells, and these transitional cells could originate from either the original form of ATII cells or SCGB3A2^+^ basal cells ^13^. Apart from this, apoptotic alveolar epithelial cells have been identified as a notable feature reported in both IPF patients or bleomycin (BLM)-induced fibrotic mice models ^14,15^. In addition, evidence supported that epithelial apoptosis was sufficient to generate fibrosis *in vivo* ^16^. The pathogenic degree of fibrosis was dependent on the apoptotic levels and could be reverted using a caspase inhibitor ^17^. Meanwhile, adjacent to fibroblast foci, co-localisation of apoptotic AECs and α-SMA marked myofibroblasts, implicating a pivotal role of dysregulated AECs during lung fibrogenesis ^18,19^. ATII cells in healthy lungs have functioned as stem cells with a renewal capacity for ATI cells ^20^. In contrast, alveolar ATII cells from fibrotic mice presented a reduced renewal capacity that could be partially associated with toll-like receptor 4 (TLR4) or Wnt/β-catenin pathway ^21,22^.

In addition to apoptosis, adjacent to the honeycombing areas from IPF lungs, hyperplastic AECs also showed a high proliferation capacity marked by proliferating cell nuclear antigen (PCNA) ^23^. These accumulated AECs were active in secreting nearly all mediators for the following ECM remodelling and disease progression. It was also reported that a small number of alveolar epithelial cells might undergo a TGFβ-mediated EMT process, expressing a range of mesenchymal related genes and enhancing collagen synthesis ^24,25^. However, lineage-tracing results from transgenic mice were inconclusive, suggesting that the proportion of these fibroblast-like ATII cells is nearly negligible ^26–29^.

Multiple risk factors such as infection, environmental exposures, occupational exposures, smoking, and genetic mutations could trigger micro-injuries to the alveolar epithelial cells and initiate fibrogenesis ^30^. However, recent evidence was found to challenge this theory. Although most evidence supported the paradigm that abnormal regeneration in IPF lungs starts with continuing exogenous micro-injuries on the AECs, researchers highlighted that alterations in the integrity of these AECs in the absence of injuries provide an alternative for IPF initiation. It has been proposed that telomere shortening in ATII cells triggered fibrogenesis. For example, in a mice model, lack of telomere repeat binding factor 1 (TRF1) in ATII cells spontaneously initiated age-associated remodelling and fibrotic responses ^31,32^. TRF1 is a type of shelterin, allowing damaged telomeres to be recognised ^31,32^. It is believed that telomerase abnormalities may result in impaired stem cell function of ATII cells so as to induce fibrosis ^33^. Consistently, persistent accumulation of the premature senescence in ATII cells has been implicated in IPF lungs ^34^. Interestingly, senescence detected in myofibroblasts inhibited fibrogenesis in many organs, including the liver, heart, and kidney ^35^. In contrast, in pulmonary fibrosis, senescence mainly occurred in the alveolar epithelial cells and was reported to be harmful ^36^. However, controversial evidence suggests that alveolar renewal failure associated with reduced telomerase integrity only predisposes patients to pulmonary fibrosis following endogenous injuries ^31^. They indicate that loss of telomeres integrity is a risk factor rather than an initial element for IPF.

In response to injuries, aberrantly activated epithelial cells secrete some pro-fibrotic regulators, forming highly contractile myofibroblasts. In such a way, aberrant alveolar epithelia contribute to the ECM deposition and disease progression. These pro-fibrotic mediators mainly comprise growth factors, matrix metalloproteinases (MMP), chemokines, coagulation factors and developmental pathways ^12,31^. Growth factors are the most predominantly secreted mediators from AECs, including TGFβ, tumour necrosis factor-α (TNF-α), osteopontin, angiotensinogen, platelet-derived growth factor (PDGF), connective tissue growth factor (CTGF), endothelin-1, and insulin-like growth factor 1 (IGF-1) ^37^. Studies implicated that these mediators are key to IPF pathogenesis as they may induce EMT, control fibroblast migration and proliferation, augment myofibroblast differentiation, and enhance ECM production. In addition, mediators from the MMP family exert their pro-fibrotic effect mainly on AECs, inducing migration and proliferation ^38^. Meanwhile, AECs may secrete several chemokines during disease progression, including CC motif chemokine ligand 2 (CCL2), CCL17 and CXC motif chemokine ligand 12 (CXCL12) ^39^. Furthermore, some of the secreted regulators are coagulation related, such as tissue factor/factor VIIa/factor X (TF/FVIIa/FX) complex, protease-activated receptor 1 and 2, and plasminogen activator inhibitor-1 (PAI-1) ^40^. Finally, it should be emphasised that the activation of several developmental pathways (*e*.*g*. Wnt and Sonic Hedgehog) could alternatively regulate fibrogenesis ^41,42^, mainly through cross-talk with TGFβ ^31,43^. Taken together, numerous epithelium-derived mediators work synergistically and antagonistically, enhancing fibroblast recruitment and activation, resulting in aberrant ECM deposition and respiratory failure ^12,31^.

### 1.2 Mesenchymal cells in IPF

In a canonical wound healing model, mesenchymal cells are implicated as the culprit for pulmonary fibrogenesis ^44,45^. Following epithelial dysfunction, several crucial processes occur, including recruitment, migration, and proliferation of fibroblasts, terminating with activation of specific contractile cells, namely myofibroblasts. Activation is typically accompanied by an increased expression of α-smooth muscle actin (α-SMA), leading to wound resolution in healthy lungs ^45^. However, in IPF, repeated stimuli trigger lung epithelial damages, inducing malfunctions in tissue repair. The fundamental problems that exist in these myofibroblasts are their anti-apoptotic properties ^45^. These activated myofibroblasts are crucial as they continuously produce large quantities of ECM components in the regions characterised as fibroblast foci, resulting in thickened interstitial tissues and eventually permanent scarring ^45^. However, the origin of these myofibroblasts is unclear and controversial. Nevertheless, recent evidence proposed 3 leading candidates, although each contribution's magnitude in the development of IPF is rarely estimated ^19^.

In mice models, bone marrow (BM) origin accounted for nearly 30%, while EMT accounted for 20% of myofibroblasts ^46^. However, the proportion of EMT may be overestimated as lineage tracing results from transgenic mice demonstrated that contribution from these fibroblast-like ATII cells is nearly negligible ^26–29^. Nevertheless, based on these mouse models, it can still be confirmed that over 50% of the activated myofibroblasts are from resident local fibroblasts ^19,46^. In IPF lungs, through the lens of scRNA-seq, an increased proportion of fibroblasts has been observed in PF compared to healthy lungs. Notably, specific fibroblast subtypes, including ACTA2^+^ myofibroblasts, a PLIN2^+^ lipofibroblast-like group, and a novel HAS1^hi^ fibroblast population, were enriched in PF lungs. These findings indicate a dysregulated fibroblast landscape in PF, with certain subtypes contributing to abnormal extracellular matrix deposition ^13^.

## 2 Epithelial-mesenchymal transition (EMT)

It was thought that resident AECs have been shown to exhibit a high degree of plasticity, meaning that they can transform into a variety of different cell types in response to different signals. This includes various types of mesenchymal cells, such as fibroblasts populations, and smooth muscle cells. in response to injury or certain pathological conditions, epithelial cells can undergo epithelial-to-mesenchymal transition (EMT). During EMT, epithelial cells lose their characteristic features such as cell polarity and cell-cell adhesion, and gain mesenchymal properties, which include increased motility and invasive capabilities. This transition allows them to become mesenchymal cells such as fibroblasts, myofibroblasts, or smooth muscle cells. Following the transition, these cells will exhibit several mesenchymal characteristics, including vimentin, fibronectin, α-SMA and N-cadherin, leading to ECM expansion ^47^. EMT occurs in response to multiple insults, including infections, ER stress, and TGFβ. In mice models, infection with gamma herpes viruses could activate TWIST-mediated EMT ^48^. Similarly, regulated by both the extracellular signal-regulated kinase (ERK) and nuclear factor kappa-light-chain-enhancer of activated B cells (NF-κB), the associations between Epstein-Barr virus (EBV) infection and EMT has also been identified ^49,50^. Furthermore, tunicamycin-induced endoplasmic reticulum (ER) stress is another cause of EMT affecting AECs ^51^. Of note, TGFβ is the most potent EMT driver ^52^, with SNAI1 demonstrated to be a key mediator ^53,54^. Overexpression of SNAI1 has been identified in AECs from IPF lungs whilst blocking SNAI expression may abolish TGFβ-induced EMT ^54^. Recent findings from our group provide additional insights into the role of EMT in IPF, where we demonstrate that the reduction of liver kinase B1 (LKB1) in epithelial cells serves as a trigger for EMT, resulting in the inhibition of autophagy and subsequent activation of the NF-kappaB (NF-κB) signaling pathway ^55–58^. This activation not only emphasizes the significance of EMT in the development of lung fibrosis but also amplifies the interaction between epithelial cells and fibroblasts in IPF. Furthermore, our research elucidates the involvement of EMT in IPF in elevating the expression of transcription factors, notably Snail2, revealing a novel aspect of the molecular mechanisms that propel the progression of IPF.

There are several studies proposed an epithelial origin of myofibroblasts within IPF lungs. For instance, AECs from IPF patients exhibited several mesenchymal markers, including α-SMA and N-cadherin ^59^. In contrast, elevated expression of epithelial-specific proteins like keratin 18 was identified in IPF myofibroblasts ^60^. Additionally, co-localisation of epithelial and mesenchymal markers from IPF lungs has been observed, including ZEB1, β-catenin, mTOR, Rho-associated coiled-coil containing protein kinase 1 (ROCK1), N-cadherin, Ki-67, vimentin, and Collagen I ^61,62^. These studies together demonstrate that AECs undergoing EMT could constitute the population of myofibroblasts. However, lineage-tracing investigations remain controversial. While some studies strongly supported the EMT-origin theory, others proposed that the proportion of epithelium-derived myofibroblasts can be neglected. It is believed that although AECs undergoing EMT exhibit some morphological changes ^63^, they remain a phenotype that is distinct from myofibroblasts ^64^. In kidney fibrosis, activation of SNAI1 was able to induce EMT ^54,65^ whilst blocking SNAI1 inhibited EMT and attenuated fibrosis ^29^. However, no evidence showed that these tubular epithelial cells directly contribute to the myofibroblast population ^65^. Instead, studies have identified an autocrine loop in which TGFβ becomes both a cause and a consequence of EMT, sustaining fibroblast to myofibroblasts transition ^54,65^. Rather than directly constituting the myofibroblasts population, EMT contributes to fibrosis mainly via mediating the microenvironment ^66^. Moreover, the available evidence points to EMT as an alternative to normal cell and tissue regeneration, potentially offering novel insights into diagnostic and prognostic biomarkers, as well as more effective treatment options for IPF ^67^. Thus, understanding the mechanisms of EMT in lung fibrosis could be utilised for developing a novel intervention for IPF.

## 3 Epithelial-mesenchymal crosstalk

IPF progression is considered as an epithelial-originated and myofibroblast-activated deterioration of the lung ^9,68^. The pathogenesis of IPF requires aberrant crosstalk between the epithelial cells and the fibroblasts. However, until now, these communications still lack further evidence and are yet to be elucidated. Factors determining why scarring occurs rather than the regular tissue repair progresses remain undefined. Once myofibroblast foci are formed, it is thought that epithelial-mesenchymal crosstalk exacerbates the progression of IPF via modulating the microenvironment. Epithelial-mesenchymal interactions create a vicious cycle in which damaged AECs enhance fibroblasts' proliferation, recruitment, and activation, resulting in aberrant ECM deposition. In return, these resultant myofibroblasts provoke epithelial injuries and promote their apoptosis. The studies on fibroblast resistance to apoptosis and increased epithelial cell apoptosis in idiopathic pulmonary fibrosis (IPF) also provide critical insights into the epithelial-mesenchymal interaction. Fibroblasts in IPF exhibit resistance to Fas-mediated apoptosis, a resistance attributed to various factors such as the increased expression of anti-apoptotic proteins and decreased levels of surface Fas. This altered apoptotic behavior is significant in the pathogenesis of IPF ^69^. On the other hand, alveolar epithelial cells in IPF exhibit an elevated inclination toward apoptosis. This heightened apoptosis of epithelial cells can hinder the effective repair and regeneration of lung tissue, thereby intensifying the fibrotic progression. The Fas-Fas ligand (FasL) pathway has been implicated in fostering pulmonary fibrosis by triggering apoptosis in alveolar epithelial cells. The presence of apoptotic epithelial cells, particularly in regions proximal to fibroblasts, has been noted in lung biopsies from patients with IPF ^70^.

These contrasting behaviours of fibroblasts and epithelial cells in IPF highlight a complex interplay of cellular mechanisms that drive the progression of the disease. Such epithelial-mesenchymal interactions are believed to exacerbate the development of many organ fibrosis, in which co-localisation of both fibroblasts and epithelial cells has been observed ^71^. Consistently, in kidney fibrosis, epithelial micro-injuries may create a pro-fibrotic microenvironment by secreting TGFβ or connective tissue growth factor (CTGF) ^72^, enhancing myofibroblast accumulation. In return, these resultant myofibroblasts induce the apoptosis of epithelial cells via angiotensin II (ANG II) or reactive oxygen species (ROS), creating a self-sustained feedback loop ^73^.

Similar crosstalk has been observed in pulmonary fibrosis. A range of pro-fibrotic mediators acting on fibroblasts derived from AECs have been strongly implicated in IPF, including multiple growth factors (*e*.*g*. TGFβ, PDGF, CTGF, TNF-α, osteopontin, angiotensinogen, and endothelin-1) and some developmental pathways (e.g. Wnt/β-catenin and Sonic hedgehog) ^74^. In response to injuries, studies found that AECs secrete a group of mediators to enhance pathogenic fibroblast functioning so that these activated AECs may contribute to ECM deposition. For instance, injured AECs stimulate TGFβ secretion, increasing fibroblast proliferation, recruitment, and activation ^75,76^. Meanwhile, secreted TGFβ1 and endothelin-1 are required to prevent fibroblast apoptosis by regulating the protein kinase B (AKT) pathway ^19,77^. In contrast, under the same microenvironment, AECs from IPF patients become more susceptible to apoptosis partially due to the decreased expression of prostaglandin E2 ^78^. In addition, our lab demonstrated that RAS-activated ATII cells provide paracrine signals to augment fibroblast activation ^79–81^. While zinc finger E-box binding homeobox factor 1 (ZEB1) knock-out mice presented with less mesenchymal gene expressions ^82^, ZEB1 is recognized as a pivotal transcription factor that governs the expression of secreted factors from AECs undergoing EMT ^79–81^. In return, protein acidic and cysteine rich (SPARC) secreted from IPF fibroblasts could disturb the integrity of the alveolar epithelium by disrupting junctional contacts ^83^. SPARC, a matrix-derived protein, is responsible for calcifying collagens, ECM production, and cell morphology alterations ^84^. While Thy-1 deficiency is a specific feature identified in IPF fibroblasts that could induce metalloproteinase-9 (MMP-9) synthesis ^85^, elevated MMP-9 may disrupt the alveolar basement membrane by inhibiting collagen IV production ^86^. Additionally, compared to media conditioned by healthy fibroblasts, conditioned media (CM) from IPF fibroblasts was able to promote the apoptosis of AECs ^71,87^. More importantly, several mediators have been identified to exert this regulatory effect, including Angiotensin II (ANG II) and hydrogen peroxide (H_2_O_2_). Angiotensin II, a key mediator found in conditioned media harvested from IPF fibroblasts ^88^, can be produced under low oxygen tension ^89^. In WT mice, both epithelial apoptosis and bleomycin-induced fibrosis can be attenuated by treating with ANG II type 1 receptor (AT1R) antagonist or antisense oligonucleotides ^90,91^. Similarly, mice with AT1R-deficiency were protected from pulmonary fibrosis ^90^. Furthermore, hydrogen peroxide (H_2_O_2_) is another fibroblast-derived mediator responsible for alveolar apoptosis ^92^. Activation of NADPH oxidase 4 (NOX4) is the leading cause of H_2_O_2_ production following lung injury^93^. While increased secretion of H_2_O_2_ correlates with IPF severity ^94^, exogenous H_2_O_2_ has proved to inhibit migration and induce the apoptosis of primary AECs ^95,96^. In a separate investigation conducted by our research team, the exploration of epithelial-fibroblast crosstalk involved co-culturing LKB1-depleted epithelial cells with MRC5 human fibroblasts, thereby reinforcing previously proposed insights. Our findings indicate that the depletion of LKB1 in ATII cells does not significantly impact collagen gene expression in 2D monocultures, despite exhibiting an EMT signature. However, in 3D co-cultures with MRC5 fibroblasts, LKB1 depletion results in a substantial upregulation of collagen genes. This suggests that the heightened collagen production in IPF is likely attributed to an indirect effect of epithelial cells on fibroblast differentiation through paracrine signaling. Additionally, the study demonstrates that LKB1 depletion enhances myofibroblast differentiation, influenced by paracrine factors and synergistically augmented by TGF-β ^55^.

**Figure 1:**
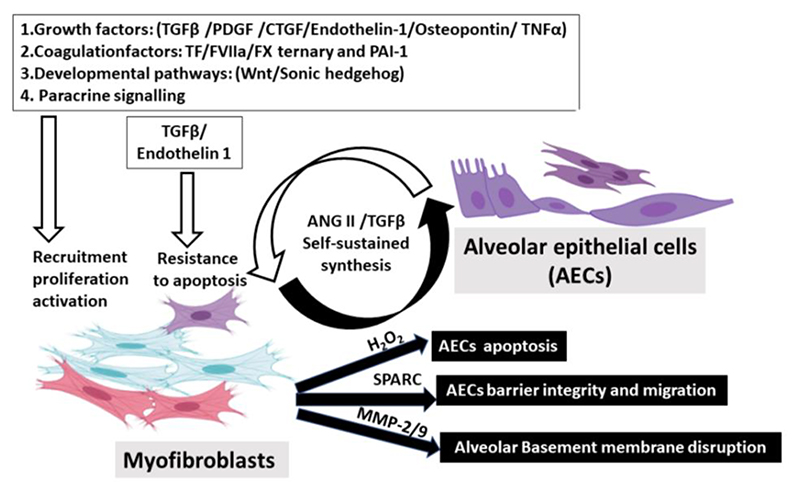
Schematic diagram of epithelial-mesenchymal crosstalk in IPF. Pathogenesis of IPF requires aberrant epithelial-mesenchymal crosstalk. In response to micro-injuries, damaged AECs will enable the secretion of many pro-fibrotic mediators, including multiple growth factors, coagulation factors, and some developmental pathway activators. These mediators act to inhibit apoptosis and stimulate migration, proliferation, and differentiation on fibroblasts. Additionally, RAS-activated AECs augment fibroblast activation by paracrine signalling so that these activated AECs are responsible for increased collagen synthesis and ECM deposition. In return, resultant myofibroblasts provoke epithelial injuries and enhance their apoptosis. Activated myofibroblasts may secrete some death-inducing mediators for epithelial cells, such as ANG II and H_2_O_2_. Furthermore, increased matrix metalloproteinases (*e.g*. MMP-9) from fibroblasts may also destroy the epithelial basement membrane by disrupting collagen IV, a key basement membrane component. Meanwhile, paracrine regulators such as SPARC from activated fibroblasts may interfere with epithelial integrity and migration. (AEC: alveolar epithelial cells; ANG: Angiotensin; CTGF: connective tissue growth factor; ECM: extracellular matrix; EMT: epithelial-mesenchymal transition; H2O2: hydrogen peroxide; MMP: matrix metallopeptidases; PAI-1: plasminogen activator inhibitor-1; PDGF: platelet-derived growth factor; SPARC: secreted protein acidic and rich in cysteine; TF/FVIIa/FX: tissue factor/ factor VIIa/ factor X; TGFβ: transforming growth factor-β; TNF-α: tumour necrosis factor-α; ZEB1: zinc finger E-box binding homeobox 1).

Importantly, several mediators exhibit bidirectionality and can be produced by both cell populations. Both ANG II and TGFβ serve as pivotal bidirectional mediators, playing a fundamental regulatory role in epithelial-mesenchymal crosstalk, influencing the microenvironment, and contributing to the aggravation of pulmonary fibrosis. As previously mentioned, fibroblast-derived ANG II induces apoptosis in AECs through paracrine signaling, while the resulting AECs also have the capacity to synthesize ANG II ^97,98^. In addition, TGFβ is a potent cytokine from AECs regulating various pro-fibrotic functions in fibroblasts ^75,76^. Recent studies suggest latent TGFβ may be produced from activated myofibroblasts in an integrin αvβ5 dependent manner ^99^, and more importantly, inducing epithelial apoptosis ^100^. Collectively, exploring the exact mechanisms involved in this epithelial-mesenchymal crosstalk is crucial to IPF pathogenesis (Figure 1).

## 4 Aberrant epithelial-mesenchymal crosstalk provides self-sustainable activation signals driving disease progression

**Figure 2:**
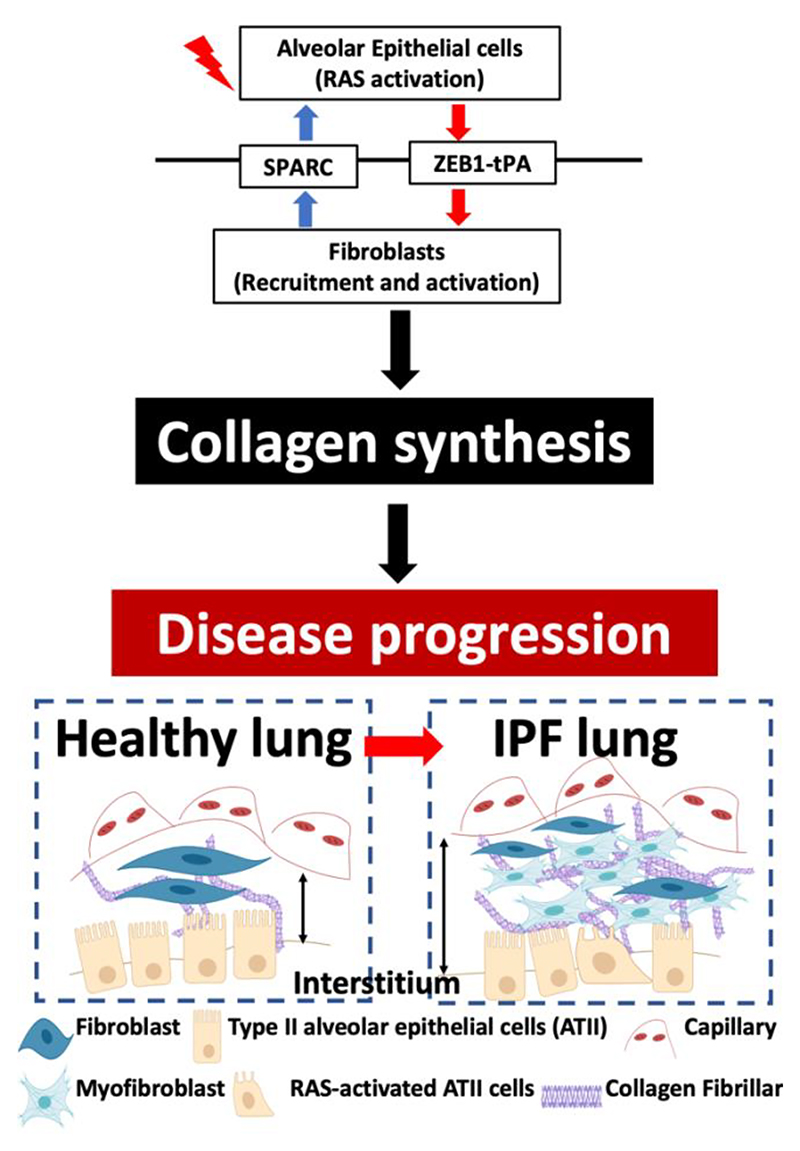
Aberrant epithelial-mesenchymal crosstalk provides self-sustainable activation signals driving disease progression. Dysregulated bi-directional epithelial-mesenchymal crosstalk activates self-sustaining pro-fibrotic signals, driving ECM deposition critical to fibrotic progression. Paracrine signalling between RAS-activated ATII cells and fibroblasts augments fibroblast recruitment and promotes TGFβ-induced activation via a ZEB1-tPA axis. Reciprocally, activated lung fibroblasts induce RAS activation in ATII cells by paracrine signalling, at least partially via SPARC. These myofibroblasts can evade apoptosis and secrete a considerable amount of fibrillar collagens, resulting in IPF progression. (IPF: idiopathic pulmonary fibrosis; ECM: extracellular matrix; ZEB1: zinc finger E-box binding homeobox 1; tPA: tissue plasminogen activator; SPARC: secreted protein acidic and cysteine rich).

Consistent with kidney fibrosis ^101^, our recent findings support the concept that aberrant epithelial-mesenchymal crosstalk contributes to the development of interstitial lung fibrosis ^66^. We establish a bi-directional profibrogenic positive feedback loop that maintains a chronic wound environment involving activated epithelial cells and fibroblasts that drive fibrosis progression rather than regular wound resolution (Figure 2). Our findings illustrate that the ZEB1-tPA axis governs paracrine signaling between RAS-activated ATII cells and fibroblasts, promoting fibroblast migration and intensifying TGFβ-induced fibroblast activation. Conversely, paracrine signaling from TGFβ-activated lung fibroblasts or fibroblasts in IPF triggers RAS activation in ATII cells, with the involvement of the secreted protein SPARC, at least to some extent.

The interacting risk factors result in micro-injuries to the alveolar epithelial cells in the lungs, leading to changes in fibroblast functionality. In response to injury, epithelial cells undergo EMT, losing their apical-basal polarity and transitioning to a migratory mesenchymal state ^47^. These resultant motile phenotypes could signal to other populations, including resident fibroblasts. We confirmed that ATII cells undergoing EMT augment TGFβ-induced profibrogenic responses in lung fibroblasts via ZEB1-mediated paracrine signalling ^79,80^. ZEB1 is strongly implicated in fibrosis, as exposure of human lungs to nickel (Ni) results in the irreversible advancement of ZEB1-dependent EMT, leading to sustained scarring in pulmonary tissue ^102,103^. We found that ZEB1 is expressed in epithelial cells of thickened alveolar septae, where ECM deposition is evident. This indicates that ZEB1 is induced as an early response to micro-injury in alveolar epithelial cells. We then demonstrated that this epithelial-mesenchymal crosstalk affects the sensitivity of TGFβ induced fibroblast activation via tissue plasminogen activator (tPA), which is a naturally secreted glycoprotein and is well recognised as a fibrinolysis activator ^104^. It possesses the capability to bind to a set of receptors through specific domains. For example, the finger domain facilitates binding to low-density lipoprotein receptor-related protein 1 (LRP1), while interaction with EGFR can occur through an epidermal growth factor-like domain ^104,105^. In a mice model of kidney fibrosis, binding to LRP1 enhances the integrin-linked kinase (ILK) dependent formation of the β1-integrin/LRP1 complex, contributing to fibroblast surveillance and fibrogenesis ^105^.

Fibroblasts from pulmonary fibrosis have altered pathogenic properties ^106^, including enhanced migration compared to fibroblasts from control lungs ^107,108^. Our research revealed that conditioned media (CM) generated by ATII cells undergoing RAS-induced EMT significantly promoted fibroblast migration. The significance of the ZEB1-tPA axis in this phenomenon was subsequently confirmed, as the silencing of these factors eliminated the pro-migratory effects of CM from RAS-activated ATII cells on lung fibroblasts. Through the regulation of tPA expression, ZEB1 may facilitate fibroblast recruitment, thereby increasing their presence at the wound site. Additionally, it has been reported that neutralizing antibodies against tPA could effectively inhibit fibroblast migration ^109^.

Importantly, TGFβ plays a crucial role in achieving paracrine effects on myofibroblast activation within the lung. In its absence, efficient fibroblast differentiation does not seem to occur even in the presence of CM from RAS-activated ATII cells. Our research indicates that the level of TGFβ receptor signaling induced by the epithelial-derived CM is insufficient to drive the differentiation of fibroblasts into myofibroblasts. These findings were further substantiated in vitro, where the addition of exogenous TGFβ was necessary to initiate myofibroblast activation. However, the upregulation of genes associated with TGFβ receptor signaling and increased expression of numerous profibrotic genes in fibroblasts, following exposure to CM from epithelial cells undergoing RAS-induced EMT, may elucidate why the conditioned media enhances the effects of exogenous TGFβ. Thus, the origin of TGFβ was investigated. In a canonical wound healing theory, Wynn and colleagues were the first to propose that after lung injury, epithelial cells release inflammatory mediators that initiate an antifibrinolytic coagulation cascade which triggers platelet activation and blood clot formation ^10^. This is followed by the entry of leukocytes that could secrete pro-fibrotic cytokines, including TGFβ ^10^, and was further reported by Kolb and colleagues ^110^. It was proposed that leukocytes may secrete IL-1β after injury to promote the production of TGFβ^110^. Potentially, RAS activation in ATII cells may release inflammatory mediators to recruit leukocytes, which then secretes TGFβ to drive fibroblast activation. This hypothesis, however, requires further investigations involving co-cultures and *in vivo* validation. Alternatively, our results further suggest that ATII cells undergoing a wound healing response could be a potential source of TGFβ, synergising with paracrine regulators for augmented fibroblast activation. Using a publicly available online LGEA portal ^111^, we found that alveolar epithelial cells from IPF lung tissue express high levels of TGFβ2 and Snail2. Previous report has proved that damaged bronchial epithelial cells were able to promote autocrine activation of EGFR whilst increasing production of TGFβ2 independent of EGFR activation ^112^. Consequently, we harvested ATII cells and observed an increased expression of TGFβ2 corresponding to the severity of the injury. The heightened expression of TGFβ2 suggests the potential involvement of the synergistic activation of underlying fibroblasts through paracrine signaling mediated by RAS-ZEB1 and Snail2-TGFβ. Thus, we propose the existence of two parallel pathways operating within these damaged and repairing epithelial cells. Some of these pathways regulate the production of profibrogenic growth factors, such as TGFβ, independently of the epidermal growth factor receptor (EGFR), while others facilitate epithelial-mesenchymal crosstalk to enhance the profibrogenic microenvironment, relying on RAS signaling.

While activation of the ZEB1-tPA axis may be a normal physiological response to injury, deregulation of this axis may sensitise the underlying fibroblasts to drive a pathogenic scarring process ^66^. In line with this notion, in studies focusing on the kidneys, micro-injuries to the renal epithelium have been suggested to establish a profibrotic microenvironment through the action of TGFβ or CTGF ^71,101^, promoting the aggregation of myofibroblasts, and in return, myofibroblasts may augment the apoptosis of epithelial cells by secreting reactive oxygen species (ROS) ^113,114^ or angiotensin II (ANG II) ^115,116^, so generating self-sustained pathogenic feedbacks. This is also true from our studies in pulmonary fibrosis. We found that fibroblasts activated by TGFβ or fibroblasts in IPF have the capacity to trigger RAS activation and induce ZEB1 expression in ATII cells through paracrine signaling. This process is, at least partially, mediated by SPARC, a cysteine-rich acidic matrix-associated protein containing three epidermal growth factor-like (EGFL) repeats. SPARC, known for its role in the calcification of collagen in bones and involvement in ECM synthesis, plays a crucial role in these interactions ^117^. We also reported that SPARC is able to enhance alveolar cell migration and dysregulate alveolar barrier integrity ^83^. In terms of mechanism, SPARC alone is capable of triggering EGFR activation in ATII cells, indicating that SPARC may signal through EGFR in a manner similar to tenascin C ^118^ via their EGFL repeats ^119^. Together, we have provided strong evidence demonstrating that in both TGFβ-activated normal lung fibroblasts and IPF fibroblasts, paracrine SPARC signalling not only dysregulates the alveolar epithelial barrier integrity ^83^ but also activates EGFR/RAS/ERK signalling in ATII cells to maintain a chronic wound-healing phenotype.

## 4 Conclusions

Dysregulated bi-directionally controlled paracrine signalling between epithelial and mesenchymal cells is a core feature of a pro-fibrotic microenvironment. EMT-related targets on epithelial cells and fibroblasts have therapeutic promise in fibrotic diseases.
